# Contributions of Body-Orientation to Mental Ball Dropping Task During Out-of-Body Experiences

**DOI:** 10.3389/fnint.2021.781935

**Published:** 2022-01-04

**Authors:** Ege Tekgün, Burak Erdeniz

**Affiliations:** Department of Psychology, Izmir University of Economics, Izmir, Turkey

**Keywords:** out-of-body experience (OBE), vestibular system, virtual reality (VR), mental ball dropping (MBD) task, full-body ownership illusion

## Abstract

Out-of-body experiences (OBEs) provide fascinating insights into our understanding of bodily self-consciousness and the workings of the brain. Studies that examined individuals with brain lesions reported that OBEs are generally characterized by participants experiencing themselves outside their physical body (i.e., disembodied feeling) (Blanke and Arzy, 2005). Based on such a characterization, it has been shown that it is possible to create virtual OBEs in immersive virtual environments (Ehrsson, 2007; Ionta et al., 2011b; Bourdin et al., 2017). However, the extent to which body-orientation influences virtual OBEs is not well-understood. Thus, in the present study, 30 participants (within group design) experienced a full-body ownership illusion (synchronous visuo-tactile stimulation only) induced with a gender-matched full-body virtual avatar seen from the first-person perspective (1PP). At the beginning of the experiment, participants performed a mental ball dropping (MBD) task, seen from the location of their virtual avatar, to provide a baseline measurement. After this, a full-body ownership illusion (embodiment phase) was induced in all participants. This was followed by the virtual OBE illusion phase of the experiment (disembodiment phase) in which the first-person viewpoint was switched to a third-person perspective (3PP), and participants' disembodied viewpoint was gradually raised to 14 m above the virtual avatar, from which altitude they repeated the MBD task. During the experiment, this procedure was conducted twice, and the participants were allocated first to the supine or the standing body position at random. Results of the MBD task showed that the participants experienced increased MBD durations during the supine condition compared to the standing condition. Furthermore, although the findings from the subjective reports confirmed the previous findings of virtual OBEs, no significant difference between the two postures was found for body ownership. Taken together, the findings of the current study make further contributions to our understanding of both the vestibular system and time perception during OBEs.

## Introduction

Out-of-body experiences (OBEs) are a type of autoschopic phenomena characterized by a sense of disembodiment (Blanke and Arzy, 2005). During OBEs, most people experience themselves in an elevated position, and this feeling is usually followed by the sensation of floating or flying localized in an extracorporeal space (Blanke et al., 2004; Bradford, 2005; Pfeiffer et al., 2014b). In the literature, OBEs were reported in various situations, including during seizures (Devinsky et al., 1989), after artificial brain stimulation (Blanke et al., 2002, 2004), and after damage to certain brain regions [i.e., the temporoparietal junction (TPJ)] (Blanke and Mohr, 2005; Blondiaux et al., 2021). Additionally, findings from the transcranial magnetic stimulation studies on the TPJ and galvanic vestibular stimulation provide further evidence for vestibular system involvement contributing to changes in visuo-spatial perspective and self-location during OBEs (Blanke et al., 2005; Lenggenhager et al., 2008). Based on these findings, it was suggested that the brain regions involved in OBEs are not only involved with vestibular processing, but are also engaged with information from different sensory modalities (Blanke et al., 2002; Ionta et al., 2011a) and related to a variety of cognitive processes, including perspective change (Palla and Lenggenhager, 2014; Deroualle et al., 2015; Pavlidou et al., 2018) and time perception (Clément, 2018; Huberle and Brugger, 2018).

Over the last decade, OBE-like experiences were also reported in healthy people (Braithwaite et al., 2011, 2013; Smith and Messier, 2014), and were experimentally induced through multisensory conflict using virtual reality techniques (Ehrsson, 2007; Lenggenhager et al., 2007, 2009; Bourdin et al., 2017). The experimental setups used during these OBE-like experiences were adapted from the original rubber-hand illusion (Botvinick and Cohen, 1998) and later used to study full-body ownership illusion (Blanke and Metzinger, 2009). Here, during a full-body ownership illusion experiment, the participants received simultaneous (synchronous) stroking to their physical body and were asked to see the visual stimulus applied to the same body location over the fake body, leading them to report an increased feeling of ownership over the fake body and to feel closer to it (Ehrsson, 2007; Lenggenhager et al., 2007). Over the years, studies employed full-body ownership illusion to study not only changes in the body-ownership but also used it to study changes in self-location (Ehrsson, 2007; Lenggenhager et al., 2007; Ionta et al., 2011b; Guterstam et al., 2015a). In fact, within the scope of the present article, a previous study by Tekgün and Erdeniz (2021) showed that full-body ownership illusion can be induced in a supine body position, providing support for the influence of vestibular signals on illusionary ownership and changes in self-location (Lenggenhager et al., 2009; Pfeiffer et al., 2014b; Pavlidou et al., 2018). Thus, it was suggested that the vestibular system and its significant role in body orientation is the main modulator of multisensory processing (Lopez et al., 2009; Lopez and Blanke, 2011; Kaski et al., 2016). This was evidenced by a wide range of experimental studies revealing that body orientation influences different aspects of bodily self-consciousness, such as perspective and self-location change (Lopez et al., 2008b, 2015; Lenggenhager et al., 2009; Thür et al., 2019; Tekgün and Erdeniz, 2021). In line with such findings, the supine body position (body-orientation in pitch axis) was shown to be associated with less accurate verticality judgments compared to sitting or standing positions (Templeton, 1973; Lichtenstein and Saucer, 1974; Goodenough et al., 1981; Tekgün and Erdeniz, 2021). This difference was supported by an early study by Saj et al. (2005), showing that patients with spatial neglect improved their performance in verticality judgments in the supine position, due to reduced asymmetrical otolith inputs. These differences are likely explained by the reduced vestibular signals available when in the supine position (Lopez and Blanke, 2010; Lenggenhager et al., 2015), similar to findings observed in microgravity environments (Lackner, 1992; Oman, 2003; Clément and Reschke, 2008; Erdeniz and Tükel, 2020; Meirhaeghe et al., 2020) and space flight analog studies, involve bed-rest (Moore et al., 2011; Koppelmans et al., 2013; Mulavara et al., 2018).

Therefore, one of the main assumptions inherent in the concept of multisensory weighting is that the supine position decreases in weight for vestibular inputs in favor of other sensory modality inputs (Lenggenhager et al., 2015). Of interest, previous studies also demonstrated that OBEs are more frequently experienced by those in the supine position (i.e., lying in bed) compared to standing (Blackmore, 1982; Irwin, 1985; Blanke et al., 2004). This difference was confirmed in around 73% of healthy individuals (Green, 1968) and 80% of patients with the neurological problem (Blanke and Mohr, 2005). Additionally, individuals' reports of the feeling of flying and floating during these experiences provided further evidence for the association between real life OBEs and altered vestibular functioning (Lopez and Blanke, 2010).

Given this evidence and the fact that OBEs occur more frequently in the supine position (Lopez and Blanke, 2010), we hypothesized that participants would show greater changes in self-location in the supine position compared to the standing position. For this purpose, in a within-group experimental design, we manipulated participants' physical body orientation (standing and supine), measuring changes in self-location before and during a virtual OBE. Similar to previous studies (Lenggenhager et al., 2009; Bourdin et al., 2017), in the current experimental setup, the participants were first introduced to a full-body ownership illusion (embodiment phase), during which a visual stimulus on the virtual body was applied synchronously with a tactile stimulus on the physical body. Following that, in the OBE phase (disembodiment phase), participants' visuo-spatial perspective switched to a third person view point, which moved to a higher location in the virtual room. Our main hypothesis on self-location was tested with a mental ball dropping (MBD) task in which the participants estimated the duration of an imaginary ball falling to the ground from their imagined location in their out-of-body experience. In this study, the MBD task allowed us not only to interpret changes in self-location during a virtual OBE but also to speculate about the changes in participants' time perception ability. Moreover, participants' subjective experiences on body-ownership and self-location were measured with a questionnaire after the embodiment and disembodiment phases. We expected an increased feeling of ownership during the former phase compared to the latter.

## Materials and Methods

### Participants

Based on studies similar to the current experimental setup (Lenggenhager et al., 2007, 2009; Aspell et al., 2009; Bourdin et al., 2017), *a priori* sample size calculation was performed for an effect size of 0.8 at 0.05 alpha level by using G^*^Power software (Faul et al., 2007). Based on that, for a one-tailed Wilcoxon signed-rank test for matched pairs (i.e., supine duration > standing duration), a required total sample size of 12 was considered necessary to reach 80% of power (Hintze, 2008). In the present study, the participants were a total of 30 volunteers (11 men, 19 women) between the ages of 19 and 39 (*Mage* = 24, *SD* = 3.93), all recruited from Izmir University of Economics. No participants reported any previous history of psychological, psychiatric, or neurological disorder, and all had normal or corrected to normal vision. Additionally, based on our demographic questionnaire, none of the participants reported experience of dizziness, ringing in the ears, vertigo, or a postural imbalance prior to the experiment. Before the experiment, the participants signed a written informed consent form and completed a questionnaire about demographic information, including their age, sex, and education levels. The present study was approved by the ethics committee of the Izmir University of Economics (No: B.30.2.IEU.0.05.05-020-066) and conducted according to the Helsinki regulations.

### Equipment and Setup

To create a wide field of view, PIMAX 5K plus head-mounted display (HMD) (https://pimax.com/about-us/) was used to present the virtual environment (200° field of view, 120 Hz). The environment was built using the game development platform UNITY 3D (https://unity.com/) version 2019.1. Two virtual characters, a male and a female avatar, were created to match the participants' gender, using Make Human software (http://www.makehumancommunity.org/). Previous studies used either real or virtual characters seen from 3PP in a dark virtual environment (Ehrsson, 2007; Lenggenhager et al., 2007, 2009) or virtual characters seen from 1PP in a virtual environment with contextual cues (i.e., virtual furniture) (Bourdin et al., 2017). In the present study, we combined the elements from the previous studies by presenting the participants with a virtual character seen from a 1PP in a dark virtual environment (refer to Supplementary Movies 1, 2). Here, the virtual environment was totally darkened, and the participants could see only their virtual body and its reflections on a full-height virtual mirror in front of them (González-Franco et al., 2010; Blom et al., 2014). Considering our focus on investigating the contribution of the vestibular system, we created the virtual characters both in the supine and standing positions, congruent with participants' physical body position. Based on that, the participants completed the experimental procedure both in the standing and supine positions on a stretcher with head supported by a yoga block to compensate for the pressure from the neck (Trousselard et al., 2003; Bringoux et al., 2018) and to minimize the proprioceptive and vestibular signals coming from the neck muscles (Mergner et al., 1997; Pettorossi and Schieppati, 2014). To account for differences in participants' height, the height of the stretcher in the supine position was calibrated to the distance of each participants' hand above the ground in the standing position (refer to Supplementary Figure 1). To animate participants' movements into virtual bodies, two HTC VIVE controllers and the Final IK asset (https://assetstore.unity.com) were used during the adaptation period of the full-body ownership illusion. To induce full-body ownership illusion, a synchronous visuo-tactile stimulation was applied with the controllers. The tactile stimulus was delivered to the abdomen of the physical body, and a spatially and temporally matched visual stimulus was seen on the corresponding location of the virtual body. OBEs were induced by manipulating the position of the virtual camera in UNITY, providing 3PP by moving the camera to an elevated position (14 m from the virtual floor) outside the virtual body, and slightly rotating it around the body x-axis, similar to the method by Bourdin et al. (2017). This perspective transformation followed a diagonal path until it reached a height of 14 m with the velocity of the camera adjusted to 0.18 m/s. Here, it is important to note that the height of 14 m was calculated not based on the relative initial camera position but is based on the absolute difference between the virtual floor and the final camera position. During the transition from 1PP to 3PP, the virtual body was stationary, but the camera rotation attached to the head of the virtual body was still under participants' control. These manipulations were based on the previous reports of OBE (Blackmore, 1982; Metzinger, 2009) and adapted from previous experimental setups (Bourdin et al., 2017). Furthermore, participants' accuracy in time perception was measured by a time reproduction task before each experimental session. For that, audacity (https://www.audacityteam.org) was used to create five auditory stimuli (sinus wave 440 Hz) with different durations (1.355, 1.916, 2.346, 2.709, and 3.029 s) which were presented through the participants' headphones. Then, changes in self-location were compared using MBD, in which the participants were asked to hold down the mouse button for the duration of the estimated time taken between the ball being released from the hand and it hitting the floor (refer to section Measurements for details). To ensure accuracy, Python 3.7 was used to record mouse button presses in both the time reproduction task and MBD task.

### Procedure

Each participant took part in two experimental sessions, which included embodiment and disembodiment phases, each including standing and supine positions. Before the experiment, it was ensured that the order of the standing and supine positions was counterbalanced, and the participants were randomly assigned to one condition. After putting on the headphones and holding the mouse in their right hands, in the standing position, the participants began the experiment with the time reproduction task, using the mouse to replicate five different fall durations with eyes closed, and without the head-mounted display.

After the headphones were retrieved, the participants were instructed about the MBD task, which they completed five times with their eyes closed in the standing position. After the mouse was retrieved, the participants were fitted with the HMD and given the VR controllers to hold. During the 1-min adaptation period, the participants were familiarized with the virtual body as they observed their head and arm movements, while their feet remained stationary (Tekgün and Erdeniz, 2021). Following that, the experimenter retrieved the controllers and returned the mouse to the participants, who were asked to wait for instructions before using it. Then, the full-body ownership illusion was induced for 1 min through visuo-tactile stimulation by tapping and stroking participants' physical body synchronously with a visual stimulation on the virtual body. During the illusion, the participants were instructed to make no bodily movements, but to focus on the visual stimulus on the virtual body by looking either directly from 1PP or in the virtual mirror reflection (Tekgün and Erdeniz, 2021).

**Figure 1:**
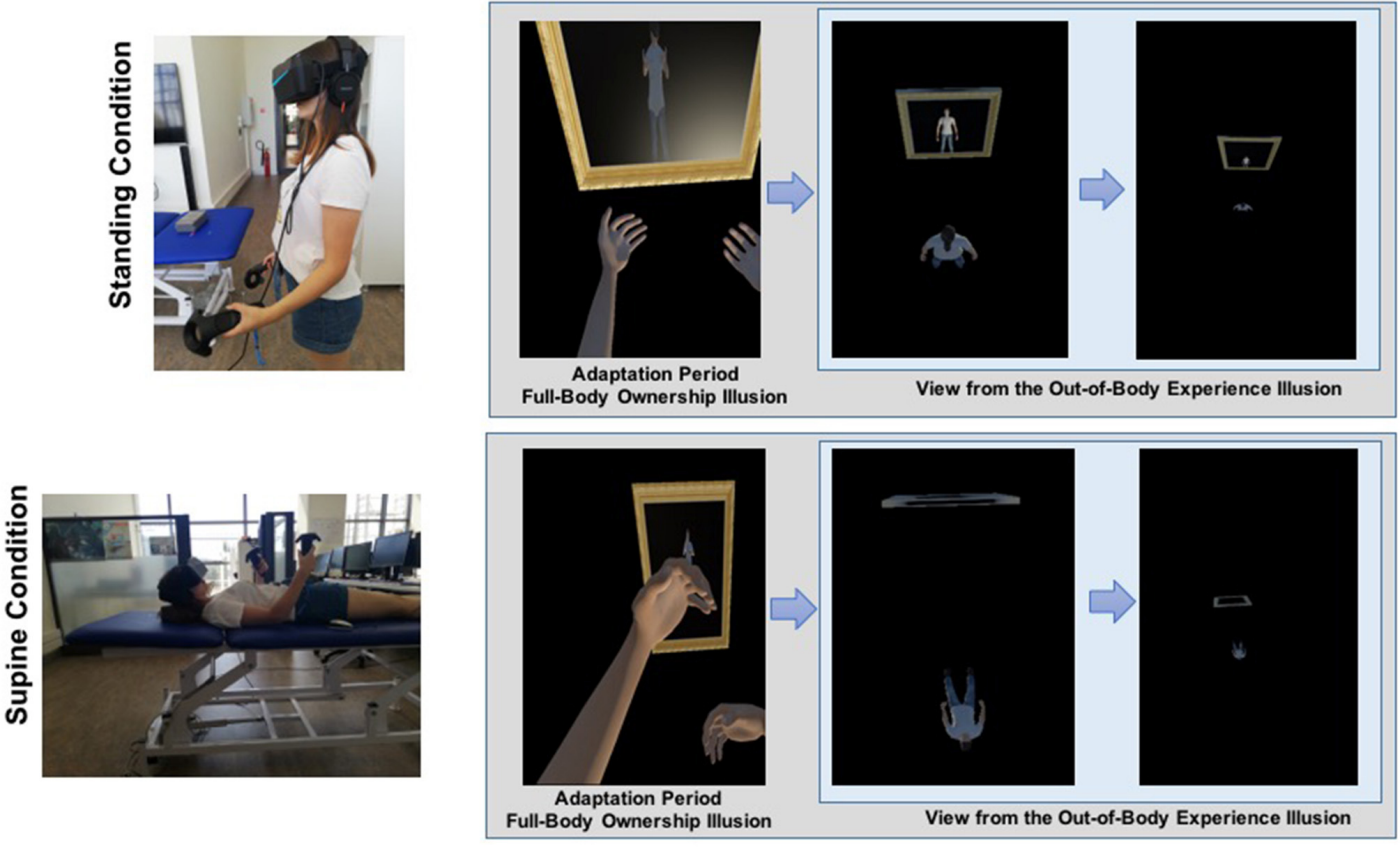
Illustration of the experimental design. **(A)** Standing condition and **(B)** supine condition. During the full-body ownership illusion, participants experience the virtual body from the first-person perspective (1PP) while being subjected to synchronous visuo-tactile stimulation. In the virtual out-of-body experiences, 1PP of participants is shifted toward up, 14 m above the virtual body.

After the full-body ownership illusion, the virtual OBE phase was initiated, in which participants' 1PP began to elevate as though gliding slowly upward, giving the impression of being 14 m above the virtual body. During this time, the virtual body was stationary, but the camera rotation was still under participants' control. When looking down, the participants saw the virtual body in a position congruent with their physical body (refer to Supplementary Movies 1, 2). After the visuo-spatial perspective transition in the OBE phase, the participants performed the MBD task five times while seeing their virtual body 14 m below them. HMD was then removed, and the participants completed the subjective report on illusory full-body ownership experiences and OBEs. The HMD was reattached, and the same experimental procedure was implemented for the other body position condition, except for the time reproduction task. After the experiment, the participants were thanked and debriefed; their questions about the experiment were answered. Figure 1 illustrates the experimental design.

### Measurements

#### Time Reproduction Task

At the beginning of the experiment, a time reproduction task was given to measure participants' time reproduction skills (Kitamuraa and Kumarb, 1984; Mioni et al., 2014). The participants listened to five different randomly presented auditory stimuli (1.355, 1.916, 2.346, 2.709, or 3.029 s) corresponding to free-fall times of an imaginary ball falling from different heights (9, 18, 27, 36, or 45 m). The durations were calculated based on the law of free fall (no air resistance) and calculated by the following equation (refer to Bratzke and Ulrich, 2021 for details):


$$\begin{array}{l} h\left(t\right)=1/2gt^{2} \end{array}$$

According to this equation, *h* corresponds to height, *t* denotes fall time, and *g* refers to acceleration factor (9.807 m/s^2^). Before listening, the participants closed their eyes and were informed that the sounds were associated with a ball dropped from a certain height. Here, the reason for emphasizing the need to reproduce the duration of a falling object is the ability of the participants to achieve accurate measurement in the time reproduction task by facilitating their mental imagery (Taatgen et al., 2007; MacPherson et al., 2009; Hargreaves, 2012), as well as to prepare them for the MBD task. However, in order to prevent the participants from learning these height-duration associations, they were not informed about the actual heights. After hearing each sound, they were asked to replicate its duration by pressing and releasing the mouse button. This measurement allowed us to note any serious impairment in time reproduction skills.

#### Mental-Ball Dropping Task

In the present study, as an implicit measure of self-location, we used MBD task in which the participants imagined the duration of the fall of the imaginary ball from their hand to the ground (Lenggenhager et al., 2009; Ionta et al., 2011b; Salomon et al., 2013; Bourdin et al., 2017). Two MBD tasks were administered in different phases of the experiment. First, in the baseline phase (*base-MBD*), the participants performed the MBD task with their eyes closed without HMD. For this measurement, it was particularly emphasized that the task should be performed from a ground level in the physical room. Following that, after the OBE phase (*obe-MBD*) in the virtual environment, the participants performed the second MBD task while watching their virtual body 14 m below them. For this measurement, particular emphasis was placed on the need for those participants to complete the task relative to their perception of the experienced ground level. In each phase, the MBD task was performed 5 times, resulting in 10 MBD measurements for each body orientation condition (supine and standing), thus in total 20 MBD measurements (10 per condition) were collected from each participant.

#### Self-Report Questionnaire

After the experiment, the participants were presented with an adapted version of the questionnaire from previous studies (Botvinick and Cohen, 1998; Lenggenhager et al., 2007). The questionnaire was presented in two parts: the first included items about full-body ownership illusion, and the second, items about OBE. The full-body ownership illusion was assessed by 2 items focusing on body ownership (*FBI1*), and self-location (*FBI2*). For the OBE phase, 5 items, such as body-ownership (*OBE1*), disembodiment (*OBE2*), vestibular sensations (*OBE3*), elevated visuo-spatial perspective (*OBE4*), and connection with the body (*OBE5*) were, respectively, tested. A paper-based questionnaire with a total of 8 items was presented on the visual analog scale (VAS) consisting of a 10 cm line with “strongly disagree” on the extreme left and “strongly agree” on the extreme right. Table 1 shows all the items in the questionnaire.

**Table 1:** The list of self-report questionnaire items for full-body ownership illusion and out-of-body experience (OBE).

**Item names**	**Item statements**
**Immediately after the time of seeing the virtual body was stroking synchronously with your physical body and reflected onto the virtual mirror**	
Ownership (FBI1)	I felt as if the virtual body was my own body.
Self-location (FBI2)	I felt as if my body was located at where the virtual body was.
**Immediately after the experience of watching the room from above**
Ownership (OBE1)	I felt as if the virtual body was my own body.
Disembodiment (OBE2)	I felt out of my virtual body.
Vestibular sensation (OBE3)	I felt as if I was floating in air.
Visuo-spatial perspective (OBE4)	I felt as if I was in an elevated position in the room.
Body connection (OBE5)	I felt a connection with the virtual body as if I was looking down at my virtual body.

### Data Processing and Statistical Analysis

Statistical analysis was performed with SPSS 20. First, to evaluate participants' time reproduction ability, and its deviations from the ideal free-fall model, we performed a linear regression analysis on the time reproduction data. Second, we performed Shapiro–Wilk-test to check for normality assumption, which showed that the data was normally distributed for the Self-Report Questionnaire but not for the MBD task. Therefore, for the MBD task, we used a non-parametric test, the one-sided Wilcoxon signed-rank test, to compare changes in *obe-MBD* and *base-MBD* times in the standing position to those in the supine position. We also computed the average changes in MBD times for each body orientation by subtracting *base-MBD* times from *obe-MBD* (Bourdin et al., 2017) and compared these using Wilcoxon signed-rank test. Thus, to test whether answers to questions differ across the standing and supine body positions, we analyzed the Self-Report Questionnaire using a paired-sample *t*-test.

## Results

### Time Reproduction Task

**Figure 2:**
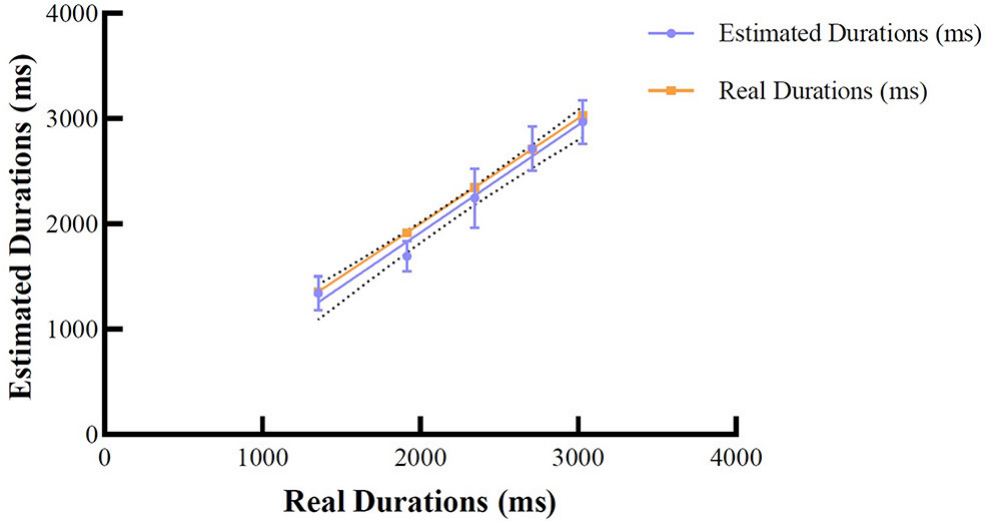
Means for the estimated durations and real durations based on the law of free fall. Error bars represent 95% CIs.

To analyze participants' accuracy of time reproduction, for each participant, we investigated estimated durations associated with the ideal free-fall time (real durations) (Bratzke and Ulrich, 2021). To achieve this, we first calculated participants' mean durations (refer to Table 2) and fitted a linear regression model with ideal durations based on the law of free fall as the predictor, and estimated durations as the dependent variable. Overall, the results demonstrated a good model fit (*R*^2^ = 0.546). The result revealed that the slopes were not significantly different, *F*_(1, 296)_ = 0.086, *p* = 0.769. That is, the means of the estimated durations were similar to the real durations, i.e., the participants were capable of reproducing time durations (pooled slope equals 1.011). Figure 2 illustrates the linear fit model for the mean of estimated durations corresponding to each real duration. The mean duration estimates for each type of auditory stimuli can be seen in Table 2.

**Table 2:** Descriptive statistics results of the time reproduction task.

**Auditory Stimuli**				
	**1355 (ms)**	**1916 (ms)**	**2346 (ms)**	**2709 (ms)**	**3029 (ms)**
Mean (ms)	1,342.20	1,692.43	2,245.80	2,713.86	2,967.50
SE (ms)	78.77	70.71	137.81	103.33	100.75

### Mental-Ball Dropping Task

**Figure 3:**
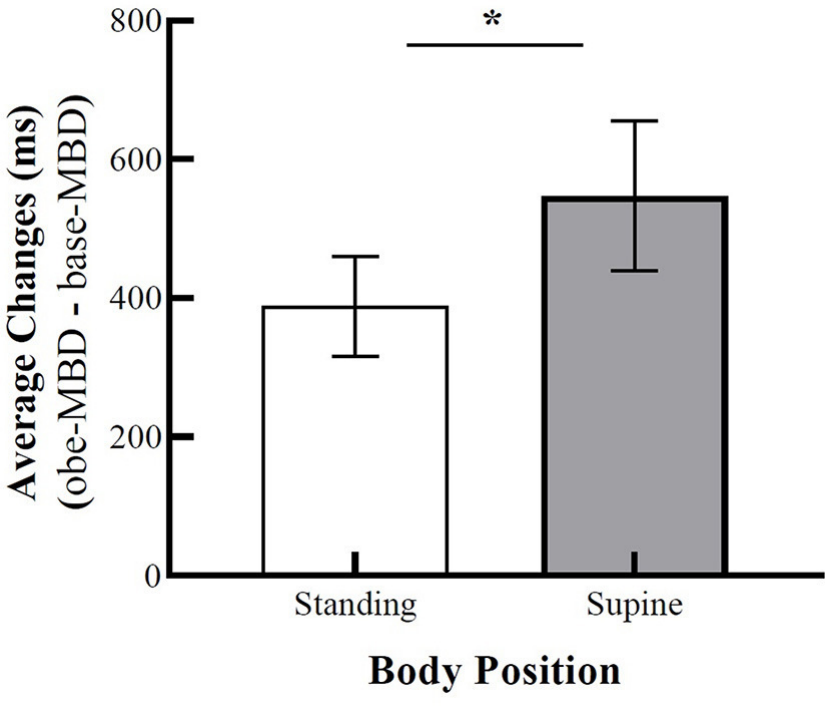
Average changes in a mental ball dropping (MBD) task between baseline and OBE phases based on standing and supine conditions. Error bars represent SEM. Asterisk denotes a significance level of *p* < 0.05.

First, to verify the effect of OBEs on the MBD task, we used Wilcoxon signed-rank test to compare MBD scores between the standing-baseline/supine-baseline and standing-OBE/supine-OBE positions. Exploration of MBD task times revealed that participants' baseline MBD times were on average 960 ms in the standing position (*Mdn* = 655.5) and 970 ms in the supine position (*Mdn* = 792), and as expected, the difference was not significant, *T* = 309.5, *z* = −1.58 *p* = 0.06, *r*_*B*_ = −0.331. Regarding the MBD times during OBEs, estimations of MBD times were on average 1,348 ms in the standing position (*Mdn* = 1,107), whereas estimations were about 1,517 ms in the supine position (*Mdn* = 1,285). The statistical analysis showed a significant difference between the standing and supine positions, *T* = 322, *z* = −2.26 *p* = 0.01, *r*_*B*_ = −0.480. Furthermore, supine-OBE and standing-OBE durations were compared against the law of free fall for 14 m that corresponds to 1,689 ms. Here, the supine-OBE (*z* = 161 *p* = 0.144, *r*_*B*_ = −0.305) position showed no significant difference from 1,689 ms. The standing-OBE (*z* = 112 = *p* = 0.013 *r*_*B*_ = −0.518) durations showed significantly shorter times than 1,689 ms. This suggests that during OBEs, the participants experienced more elevated self-location, as measured by MBD response times, in the supine position as compared to the standing position. A figure representing the results is available in Supplementary Figure 3. To clearly show the shift in self-location, we analyzed the difference between *base-MBD* times and *obe-MBD* times for each body position using Wilcoxon signed-rank test. The changes in MBD times in the supine position were on average 547 ms (*Mdn* = 347) compared to an average of only 388 ms (*Mdn* = 215) in the standing position. The statistical analysis revealed that the changes in MBD estimations were significantly smaller in standing compared to supine positions, *T* = 319, *z* = −1.78, *p* = 0.038, *r*_*B*_ = −0.372 (Figure 3).

### Self-Report Questionnaire

**Figure 4:**
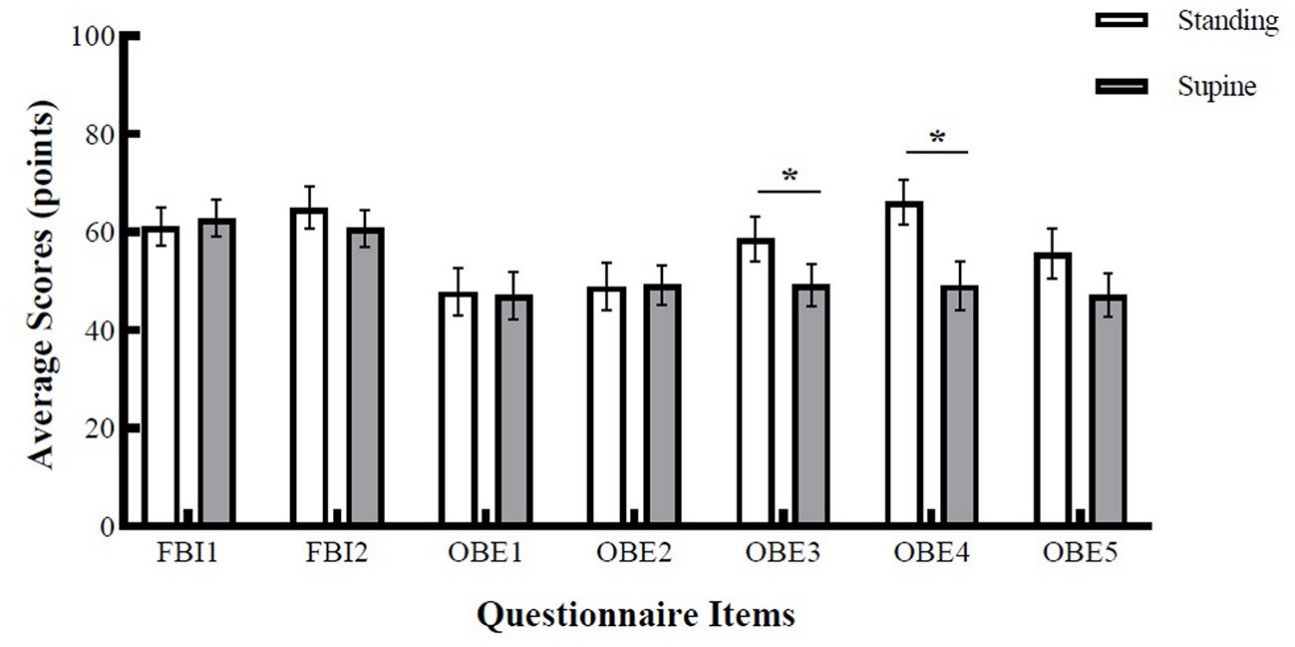
Average scores of the self-report questionnaire. Error bars represent SEM. Asterisk denotes a significance level of *p* < 0.05.

Figure 4 shows the results of paired sample *t*-test. For items related to full-body ownership illusion, there was no significant difference between the standing and supine positions. On average, participants' ratings for body-ownership (*FBI1*) in the standing position (*M* = 61.1, *SE* = 3.86) were not statistically different from the ratings in the supine position (*M* = 62.8, *SE* = 3.77), *t*_(29)_ = −0.433, *p* = 0.67. For ratings on self-location (*FBI2*), the difference between standing (*M* = 64.9, *SE* = 4.3) and supine positions (*M* = 60.8, *SE* = 3.73) was also non-significant, *t*_(29)_ = 0.11, *p* = 0.23. During OBEs, the participants reported similar body-ownership (*OBE1*) experience in standing (*M* = 47.8, *SE* = 4.83) and supine positions (*M* = 47.1, *SE* = 4.77), the difference was non-significant, *t*_(29)_ = 0.11, *p* = 0.46. The difference of disembodiment ratings (*OBE2*) between standing (*M* = 48.9, *SE* = 4.96) and supine positions (*M* = 49.2, *SE* = 4.04) was also non-significant, *t*_(29)_ = −0.04, *p* = 0.52. However, two of the items related to OBE phase revealed significant differences between standing and supine positions. The results for vestibular sensations (*OBE3*) revealed a significant difference between standing and supine positions, *t*_(29)_ = 1.84, *p* = 0.04, *d* = −0.37. That is, participants reported stronger vestibular sensations during OBE phase in the standing position (*M* = 58.6, *SE* = 4.61) compared to the supine position (*M* = 49.2, *SE* = 4.34). Similarly, the experience of elevated visuo-spatial perspective (OBE4) was stronger in the standing position (*M* = 66.1, *SE* = 4.47) compared to the supine position (*M* = 49.1, *SE* = 4.98), and the difference was statistically significant, *t*_(29)_ = 2.40, *p* = 0.01, *d* = 0.44. Rating for body connection item (OBE5) revealed that feeling of connection with the body was non-significant between the two positions, *t*_(29)_ = 1.68, *p* = 0.052. Participants rated OBE5 item on average with 55.7 points (*SE* = 5.09) in the standing position and 47.1 (*SE* = 4.49) in the supine position.

Additionally, to verify that full-body ownership illusion induced the illusory ownership of the virtual body, we used paired sample *t*-tests to compare the ratings of ownership items after the illusion with those after OBE, in both standing and supine positions. The results showed that participants experienced stronger feeling of ownership following the illusion both in the standing position, *t*_(29)_ = 3.08, *p* = 0.005, *d* = 0.562, and supine position, *t*_(29)_ = 2.63, *p* = 0.014, *d* = 0.48. On average, ownership ratings after the full-body ownership illusion were 14.48 points higher than the ratings after OBE, suggesting a successful induction of the illusion of body ownership and mild disembodiment during OBE as observed in the literature (Bourdin et al., 2017). A figure representing the results can be found in Supplementary Figures 2, 3.

## Discussion

Blanke and Mohr (2005) suggested that OBEs are characterized by three different subjective experiences: “the feeling of being outside one's physical body (or disembodiment); the presence of a distanced and elevated visuo-spatial perspective (or perspective); and the seeing of one's own body (or autoscopy) from this elevated perspective” (p. 186). On the basis of this characterization, we investigated the influences of the body-orientation on OBEs with a virtual OBE illusion set-up inspired by previous studies (Ehrsson, 2007; Lenggenhager et al., 2007, 2009; Bourdin et al., 2017). This was achieved by manipulating the physical body position and by comparing time estimations in the MBD task as an objective measure for quantifying the changes in self-location during the OBE illusion. Here, it is important to remember that the fundamental purpose of including the supine position was to create a condition with decreased vestibular input, and thus, bring about a modification of the sensory weighting strategies (Lopez and Blanke, 2010; Tekgün and Erdeniz, 2021), which might potentially increase the durations recorded in this position. In order to achieve this, first, the participants performed a time reproduction task with different durations corresponding to free-fall times of an imaginary ball falling from different heights. The results of this task showed that participants, on average, can successfully reproduce these durations and the mean durations have a good model fit based on the ideal free fall model. Furthermore, in order to explore the subjective changes in OBE, we adapted the questionnaire of Bourdin et al. (2017). The results of the questionnaire on body ownership showed no significant difference between the supine and standing positions, either during the full-body ownership illusion phase or during the out-of-body phase. However, a comparison of the main effect of ownership before (FBI1) and after the out-of-body phase (OBE1) (regardless of the body position) showed a significant fall in the body ownership scores for both conditions. This finding provides support for the successful induction of the sense of embodiment during full-body ownership illusion, which was lost during the OBE phase. Regarding our initial hypothesis related to self-location, the data from the MBD task showed increased MBD durations in the supine position compared to the standing position, suggesting greater changes in self-location in the former position. In summary, these results extend earlier findings regarding a stronger out-of-body experience in the supine position, and we discuss below possible explanations and alternative theoretical frameworks underlying these.

The first explanation related to increasing in MBD duration in the supine position is highlighted in studies showing that the vestibular system is involved in a multitude of functions, including spatial orientation (Brandt, 1999; Clément and Reschke, 2008; Clemens et al., 2011), perspective-taking (Deroualle et al., 2015; Lopez, 2016), mental imagery (Falconer and Mast, 2012; van Elk and Blanke, 2013), and time perception (Davis et al., 2009; Kaski et al., 2016). This explanation is based on the theory that the brain regions involved in OBEs and MBD have common neural origins in the TPJ and surrounding brain areas, covering parieto-insular areas (Blanke et al., 2004; Ionta et al., 2011b; Shinder and Newlands, 2014; Smith and Messier, 2014; Lopez and Elzière, 2018; Rousseau et al., 2019; Blondiaux et al., 2021). Here, it is important to note that, despite the debate over the exact cortical location of the vestibular system (Lopez and Blanke, 2011; Lopez et al., 2012; Frank and Greenlee, 2018), from its cortical interaction, it is considered that vestibular and other sensory signals with respect to the body orientation are likely to draw on shared neural resources, as well as computations carried out by adjacent or overlapping brain regions (Van Beuzekom and Van Gisbergen, 2000; Zupan et al., 2002; MacNeilage et al., 2006; Vrijer et al., 2008). In fact, a study by Kaski et al. (2016) showed that patients with TPJ lesions have impaired deficits in time estimation (motion duration) and position perception. It can be argued that different mental functions (i.e., mental imagery, spatial orientation, and timing) might share neural resources and that decreased vestibular input might lead to increased computational resources for other functions (Walsh, 2003; Huberle and Brugger, 2018), potentially increasing the estimated durations during the supine position. Referring to our results, MBD time during supine-OBE showed no significant difference from the ideal free fall estimate, while standing-OBE showed significantly shorter durations. According to the above explanation, one can argue that during supine OBE, the vestibular system (which is idle) and other related sensory systems can make more accurate estimations on the temporal changes related to bodily movements (Lacquaniti et al., 2015), by encoding the changes in the body and head movements in relation to gravity. This could then provide increased information about self-motion during the supine OBE, which could contribute to the accuracy of estimations regarding the changes in self-location and self-orientation in space (Seemungal, 2014). Therefore, in relation to its specific role in calculating the internal model of gravity, it can be argued that the vestibular system plays an important role in estimating the timing of spatiotemporal actions (McIntyre et al., 2001; Zago et al., 2004; Zago and Lacquaniti, 2005), even during virtual OBEs. In fact, there is evidence for this explanation from functional neuroimaging and transcranial magnetic stimulation studies showing that several areas in temporoparietal regions covering TPJ are involved in OBE (Blanke and Mohr, 2005; Blondiaux et al., 2021) and in time perception (Indovina et al., 2005, 2013; Bosco et al., 2008; Miller et al., 2008; Lacquaniti et al., 2013, 2015; Kheradmand et al., 2015). Indeed, it was shown that time duration estimations were impaired by both vestibular stimulation (Capelli et al., 2007) and weightlessness (Semjen et al., 1998).

Another line of research suggests that body orientation manipulation might also change the internal model gravity (Van Beuzekom and Van Gisbergen, 2000; Lopez et al., 2009), possibly tying it to a coordinate system (i.e., 3PP) other than egocentric coordinates (Moscatelli and Lacquaniti, 2011). Accordingly, in the present study, in the standing position, the gravitational up and bodily up were aligned, but in the supine position, they were orthogonal. In that case, weighting more on vision to resolve the conflict in the supine position might then lead to a stronger experience of being located at the 3PP, and this could explain the longer time estimations in the MBD task. This argument is supported by previous studies showing disruption in the normal time course of representational gravity when the body is not aligned with the environmental gravity axis (de sá Teixeira et al., 2017). For example, it was shown that participants produce longer temporal duration in 0 g compared to 1 g environments (Clément, 2018). All these studies suggest that orientation perception relative to the external environment alters the uncertainty regarding the direction of “down,” with a potentially significant effect on the MBD duration estimations (de sá Teixeira, 2014; de sá Teixeira and Hecht, 2014; de sá Teixeira et al., 2017).

Further possible explanations for the current findings involve two changes that occur during OBEs: (i) in self-location (“Where am I in space?”) and (ii) in perspective (“From where do I perceive the world?”). According to this explanation, during OBEs, participants in the supine position might experience an increased feeling of altitude (i.e., change in self-location), which might then lead to longer MBD durations. Evidence for this is provided by previous studies showing that self-location and 1PP are intricately connected (Maselli and Slater, 2013; Pfeiffer et al., 2014a; Guterstam et al., 2015b), and therefore, the definition of self-location was extended further to address the collective contribution of body-location and location of 1PP (Huang et al., 2017). In this relationship, the vestibular system is considered as a core binding mechanism that critically maintains the integrity between visuo-spatial perspective and the body (Lopez et al., 2008b; Lopez and Blanke, 2010), and indirect evidence for such a relationship is also seen in the influence of artificial vestibular stimulations on tasks that require the mental rotation of one's own body (Mast et al., 2006; Lenggenhager et al., 2008; Falconer and Mast, 2012; van Elk and Blanke, 2013). Accordingly, during the supine OBE, the participants might experience a greater disruption in relations between self-location and visuo-spatial perspective, possibly leading to the increased feeling of altitude and longer MBD durations. According to this explanation, multiple brain areas, one of which is the hippocampus, may be jointly responsible for coding self-location (Guterstam et al., 2015a,b, 2020), and also, for coding the relation between time and distance (Kraus et al., 2013), and this signal may also be integrated into the parieto-insular areas, possibly including TPJ (Craig, 2009; Wittmann, 2009). Further evidence is also provided by studies showing that changes in self-location can affect distance estimations (Harris and Mander, 2014).

An alternative, and more plausible explanation, is that spatial representations for computing time are affected by the changes in the visual perspective (i.e., egocentric and allocentric). Previous studies showed that TPJ activity was not only modulated by the visuo-tactile synchrony of stroking, but was also, differently influenced by perspective-taking (Slater et al., 2010; Ionta et al., 2011a). This aligns with a previous study suggesting that perspective-taking is a strongly embodied process and that longer reaction time may relate to the incongruence between the posture of the participant's actual body and that of a distant avatar (Kessler and Rutherford, 2010; Kessler and Thomson, 2010; Deroualle et al., 2015). Further evidence is provided by a series of behavioral studies, in which Kessler et al. showed that participants were more ready to adopt the viewpoint of an avatar when it matched their body posture (Kessler and Rutherford, 2010; Kessler and Thomson, 2010). This line of research emphasizes the key role of the switch from 1PP to 3PP, suggesting that the vestibular signal might disintegrate when the contribution from 1PP is lost (Brugger et al., 1997; Blanke et al., 2002, 2004; Blanke and Mohr, 2005; Lopez et al., 2008b; Lopez and Blanke, 2010; Ionta et al., 2011b). This interpretation is consistent with the perceived self-motion and perceived self-orientation function of the vestibular system (Kaski et al., 2016). Taken together, these results could explain the increased estimations of time following the change from the 1PP to 3PP in the supine position.

Questionnaire results showed that for both conditions, the strong body ownership illusion during the embodiment phase (FBI1) became weaker during the OBE phase (OBE1). Moreover, the disembodiment question (OBE2) also showed a significant decrease for both standing and supine conditions during OBE but with no significant difference between the two conditions. This emphasizes the possibility that the change from the egocentric viewpoint to 3PP may have created similar amounts of dis-ownership over the virtual body during OBE for both body positions. In fact, this finding is in line with the studies suggesting that OBEs are not directly characterized by complete dis-ownership of the physical body, but rather, by the localization in and the attribution of the self to an illusory body, which corresponds to the particular perspective (Lopez et al., 2008a). Moreover, the questionnaire results regarding vestibular sensations (OBE3) and elevated visuo-spatial perspective (OBE4) showed that participants reported stronger vestibular sensations and more elevated visuo-spatial perspective in standing compared to the supine body position. However, as we discussed earlier, the MBD task indicated that participants experienced being more elevated in the supine compared to the standing position. As further discussed below, this apparent contradiction might be associated with the management of sensory information (i.e., vestibular and proprioceptive). Accordingly, for the MBD task results, the experience of being more elevated in the supine position might be explained by the decrease in vestibular and proprioceptive signals (i.e., decreased input from the feet, and the more relaxed muscles). According to this argument, the decrease in vestibular and proprioceptive signals might possibly increase weight in vision, resulting in the stronger experience of being in the location of the visuo-spatial perspective, as indicated by longer estimation times. This finding is supported by the recent study by Beauchet et al. (2018), showing that the supine position is associated with more accurate mental chronometry. They argued that the decrease of vestibular and proprioceptive signals in the supine position might enhance the mental imagery process, possibly leading to more accurate duration estimations. Overall, we suggest that longer estimated times in the supine position compared to the standing position may stem from the absence of interfering vestibular and proprioceptive signals, leading to a greater reliance on vision, allowing for a focus on mental imagery during the MBD task. However, regarding vestibular sensations (OBE3) and elevated visuo-spatial perspective (OBE4) questions, stronger vestibular sensations and elevated visuo-spatial perspective in the standing position might be equally well-explained by the active perception of orientation during standing (Peterka, 2002). According to this argument, in the standing position, unlike in the supine position, the constant force experienced from the ground serves to stabilize and maintain body orientation. The body, therefore, is not motionless but generates compensatory actions based on the information from lower body parts (Stoffregen and Riccio, 1988). As a result, one possible interpretation for the questionnaire findings might be related to the involvement of the additional proprioceptive information available while standing. Such that, when standing, active proprioceptive stimulation may overcome vestibular uncertainty (i.e., about the elevation during OBE when the physical bodies were in the standing position), and thus, the sense of visually perceived elevated perspective is enhanced with the sensation of floating. Similarly, there is also the possibility that the natural tendency to sway and lean in the standing position may have enhanced sensitivity to graviception, resulting in increased vestibular sensations.

Finally, as mentioned above, it is also important to note that the vestibular system is closely associated with the perception of self-motion and spatial orientation (Day and Fitzpatrick, 2005; Angelaki et al., 2009; Fetsch et al., 2009; Pfeiffer et al., 2014b). Accordingly, several studies revealed the contribution of the vestibular system to the spatial aspect of bodily self-consciousness, specifically, to the egocentric viewpoint (Ionta et al., 2011b; Pfeiffer, 2015; Pavlidou et al., 2018; Deroualle et al., 2019). For example, Pavlidou et al. (2018) clearly showed that vestibular stimulation boosts the egocentric viewpoint, and if this is the case (Peterka, 2002; Chiba et al., 2016; van Kordelaar et al., 2018), during standing, the active vestibular system might attempt to maintain the egocentric perspective based on the point of view of the physical body during OBE phase, and as a result of this mismatch (i.e., similar to motion sickness), participants might experience stronger vestibular sensations and elevated visuo-spatial perspective in the standing position.

### Limitations

The findings of the current study are naturally subject to some limitations. First, previous studies showed the close relationship between the perception of time and space (Glicksohn, 1992; Kraus et al., 2013; Lacquaniti et al., 2015; Clément, 2018; Huberle and Brugger, 2018), and that the perception of time is significantly influenced by scale model environments and altered sensory environments (Glicksohn, 1992; Riemer et al., 2014; Mitchell and Davis, 2016; Glicksohn et al., 2017). Thus, in the current study, the deliberate use of a dark virtual environment without boundaries (i.e., walls) might have significantly influenced the results. For future research, it is important to replicate the current findings in different model environments. Second, despite calibrating the height of the bed to match the participant's hand distances from the floor in the supine posture, the calibration might have been inaccurate, and participants might have confused their height from the floor with their height from the stretcher. Thus, although there is no indication of such a scenario based on the baseline MBD durations, caution is needed when replicating our experimental design and using height-adjustable stretchers. Finally, a few previous studies have shown that the participant's posture relative to gravity direction contributes to a sense of “upwards” and “downwards” during the calculation of gravitational motion (Senot et al., 2005; Le Séach et al., 2010; Baurès and Hecht, 2011). In the current study, during the virtual OBE illusion phase, participants were able to freely move their head “upwards” or “downwards,” as well as sideways, and we did not control for such gravitational direction effects. For future research, while creating virtual OBE illusion, it is important to include a control condition with a fixated direction of gaze (i.e., looking upward to the ceiling or downward to the virtual body) to eliminate any gravitational direction effect.

## Conclusion

In the current study, it was shown that virtual OBE illusion can be induced both during standing and supine body positions. The present data, based on participants' subjective reports, showed no significant differences between the two positions in terms of the feeling of disembodiment. Moreover, the subjective reports revealed stronger feelings of floating and elevation in the standing position, although it should be noted that the results of the implicit measurement suggest that longer MBD durations were often experienced in the supine posture. Thus, the results of the current study, we believe, provide important insight into the understanding of vestibular contributions on experiencing OBEs, as well as time perception during OBEs.

## Data Availability Statement

The raw data supporting the conclusions of this article will be made available by the authors upon request, without undue reservation.

## Ethics Statement

The studies involving human participants were reviewed and approved by Ethics Committee of the Izmir University of Economics (No: B.30.2.IEU.0.05.05-020-066). The patients/participants provided their written informed consent to participate in this study.

## Author Contributions

ET and BE contributed equally to conception, design, statistical analysis and writing of the first draft of the manuscript. All authors contributed to manuscript revision, read, and approved the submitted version.

## Funding

This study was supported by a grant from TUBITAK Research (119K807).

## Conflict of Interest

The authors declare that the research was conducted in the absence of any commercial or financial relationships that could be construed as a potential conflict of interest.

## Publisher's Note

All claims expressed in this article are solely those of the authors and do not necessarily represent those of their affiliated organizations, or those of the publisher, the editors and the reviewers. Any product that may be evaluated in this article, or claim that may be made by its manufacturer, is not guaranteed or endorsed by the publisher.
