# Supplementary material for: Contributions of Body-Orientation to Mental Ball Dropping Task During Out-of-Body Experiences
Source: Front Integr Neurosci. 2022 Jan 4;15:781935. doi: 10.3389/fnint.2021.781935 (PMC8764241; doi:10.3389/fnint.2021.781935)
Supplement: Supplementary file 1 [file Data_Sheet_1.docx]

Supplementary Material


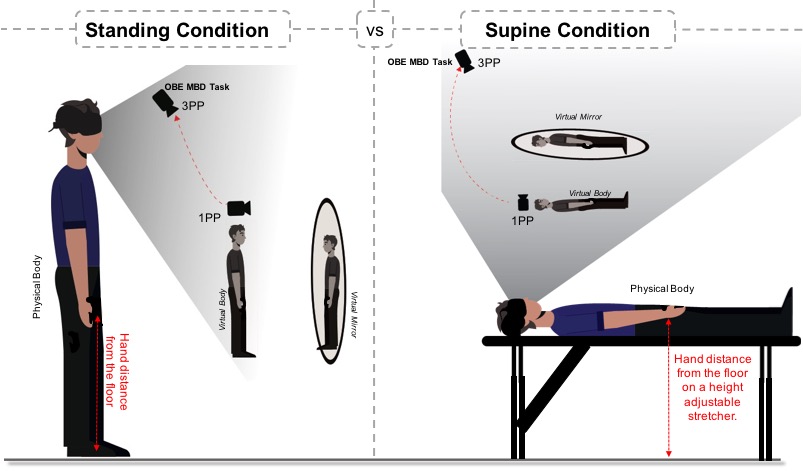


**Supplementary Figure 1.** Figure shows the camera transitions for the standing and supine conditions.


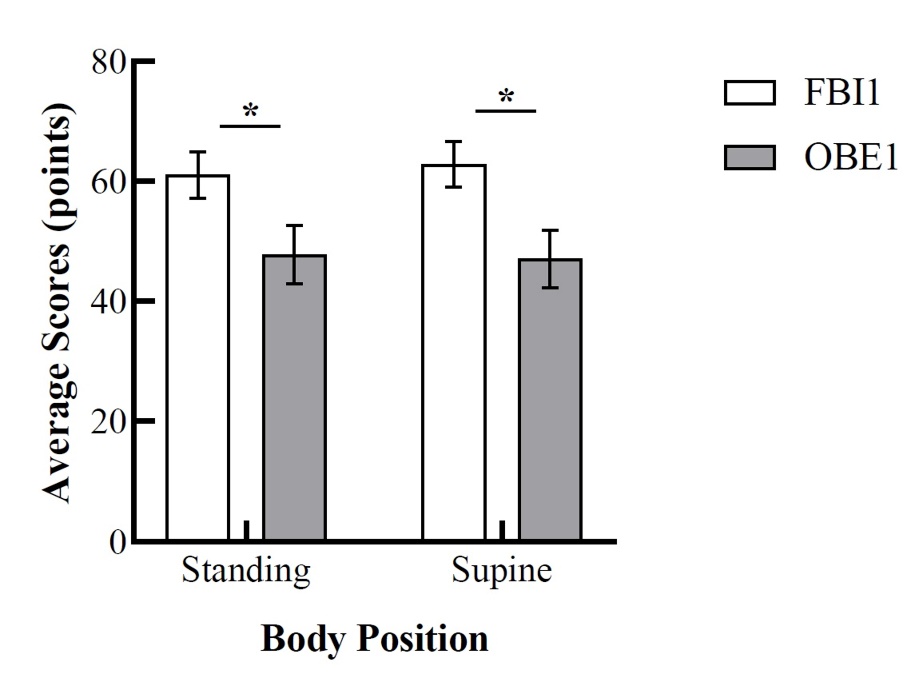


**Supplementary Figure 2.** Average scores for the ownership questionnaire items following full-body ownership illusion (FBI1) and after the OBE (OBE1) during standing and supine conditions. Error bars represent standard error of the mean.


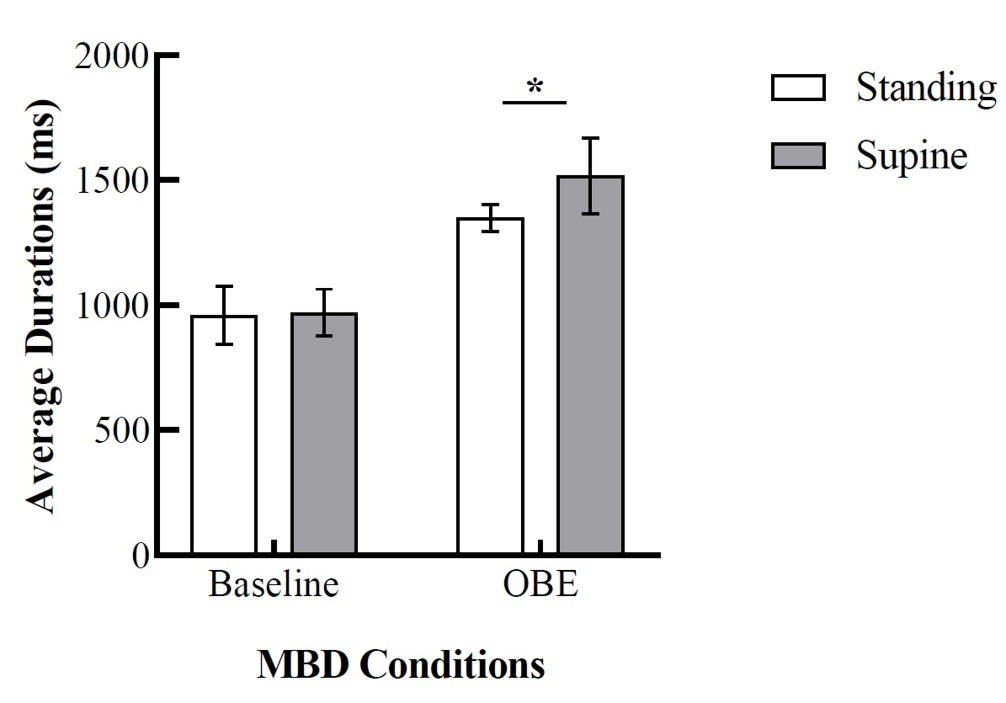


**Supplementary Figure 3.** Average duration estimations during MBD task for baseline and OBE measurements in standing and supine conditions. Error bars represent standard error of the mean.

**Supplementary Movie 1.** Movie shows a portion of the virtual OBE during standing condition, in which participants’ 1PP began to elevate as though gliding slowly upwards, giving the impression of being above the virtual body. During this time, the virtual body was stationary, but the camera rotation was still under participants’ control.

**Supplementary Movie 2.** Movie shows a portion of the virtual OBE during supine condition, in which participants’ 1PP began to elevate as though gliding slowly upwards, giving the impression of being above the virtual body. During this time, the virtual body was stationary, but the camera rotation was still under participants’ control.
